# ZNF198 Stabilizes the LSD1–CoREST–HDAC1 Complex on Chromatin through Its MYM-Type Zinc Fingers

**DOI:** 10.1371/journal.pone.0003255

**Published:** 2008-09-22

**Authors:** Christian B. Gocke, Hongtao Yu

**Affiliations:** Howard Hughes Medical Institute, Department of Pharmacology, The University of Texas Southwestern Medical Center, Dallas, Texas, United States of America; University of Washington, United States of America

## Abstract

Histone modifications in chromatin regulate gene expression. A transcriptional co-repressor complex containing LSD1–CoREST–HDAC1 (termed LCH hereafter for simplicity) represses transcription by coordinately removing histone modifications associated with transcriptional activation. RE1-silencing transcription factor (REST) recruits LCH to the promoters of neuron-specific genes, thereby silencing their transcription in non-neuronal tissues. ZNF198 is a member of a family of MYM-type zinc finger proteins that associate with LCH. Here, we show that ZNF198-like proteins are required for the repression of E-cadherin (a gene known to be repressed by LSD1), but not REST-responsive genes. ZNF198 binds preferentially to the intact LCH ternary complex, but not its individual subunits. ZNF198- and REST-binding to the LCH complex are mutually exclusive. ZNF198 associates with chromatin independently of LCH. Furthermore, modification of HDAC1 by small ubiquitin-like modifier (SUMO) *in vitro* weakens its interaction with CoREST whereas sumoylation of HDAC1 stimulates its binding to ZNF198. Finally, we mapped the LCH- and HDAC1–SUMO-binding domains of ZNF198 to tandem repeats of MYM-type zinc fingers. Therefore, our results suggest that ZNF198, through its multiple protein-protein interaction interfaces, helps to maintain the intact LCH complex on specific, non-REST-responsive promoters and may also prevent SUMO-dependent dissociation of HDAC1.

## Introduction

The ordered assembly of genomic DNA into a proteinacious substance—chromatin—allows for high-order regulation of DNA-templated processes, such as transcription, replication, and DNA repair. Chromatin contains repeating units of nucleosomes, which consists of one histone H3/H4 tetramer and two H2A/H2B dimers wrapped around by double-stranded DNA [1]–[3]. Polymers of nucleosomes flanked by various lengths of linker DNA can fold into compacted high-order structures that are subject to dynamic regulation [4].

Post-translational modifications on the flexible tails of histones can directly or indirectly affect chromatin structure [5]. Histone acetylation is generally associated with transcriptional activation and is dynamically regulated by histone acetyltransferases (HATs) and histone deacetylases (HDACs) [5]. The effect of histone lysine methylation, catalyzed by methyltransferases, depends on the specific residue and degree of modification (mono-, di-, or trimethylation) [6]. Histone H3 lysine 4 di- and trimethylation (H3K4me2/3) is associated with active promoters [7], [8], but H3K9me2/3 is mostly associated with transcriptional repression [9]. Lysine-specific demethylase 1 (LSD1; also known as BHC110 or AOF2) is a flavin adenine dinucleotide (FAD)-dependent amine oxidase that demethylates histone H3K4me1/2, but not H3K4me3 [10], [11]. Although LSD1 alone can demethylate bulk histones or peptide substrates, it requires a co-factor, REST co-repressor (CoREST), for efficient binding to nucleosomes and demethylation of nucleosomal substrates [12]–[14]. The LSD1–CoREST interaction also stabilizes the LSD1 protein in the cell [14]. A fraction of the abundant class I HDACs, HDAC1 and HDAC2, associate with LSD1–CoREST, forming an LSD1–CoREST–HDAC1/2 (LCH) core ternary complex [15]–[18]. Formation of this complex on chromatin enables HDAC1/2 and LSD1 to stimulate each other's activity through CoREST [19].

The LCH complex can be targeted to specific promoters through binding to sequence-specific transcriptional factors, either directly or indirectly. For example, RE1-silencing transcription factor (REST), which is a Krüppel-like zinc finger-containing protein, binds directly to CoREST and recruits the LCH complex to neuron-specific gene promoters that contain RE1 elements, thus repressing the expression of neuron-specific genes in non-neuronal tissues [20], [21]. In addition, LCH can be incorporated into a larger co-repressor complex that also contains CtBP1/2 and the G9a histone H3K9 methyltransferase [16]. CtBP1/2 in turn binds to Krüppel-like zinc finger-containing sequence-specific repressors ZEB1/2, which recruit this complex to chromatin [22]. Finally, LSD1 is targeted to androgen- and estrogen-responsive promoters through interactions with androgen receptor (AR) and possibly estrogen receptor (ER). In this context, LSD1 activates transcription through promoting the demethylation of H3K9me1/2 at these promoters [23], [24]. Whether the entire LCH complex is targeted to AR- or ER-dependent promoters is unclear.

Several human proteins, including ZNF198, ZNF237, ZNF261, ZNF262, and ZNF258, contain a stretch of unique tandem zinc fingers called MYM (myeloproliferative and mental retardation) domains [25] (Figure S1). The MYM-domains of ZNF198 are frequently fused to FGF receptor kinase in myeloproliferative syndromes [26]–[28]. Disruptions near the ZNF261 gene have been linked to X-linked mental retardation [29]. Among human MYM-domain proteins, ZNF198, ZNF261, and ZNF262 share a similar domain architecture and possibly perform similar functions (Figure S1). A *Drosophila* homolog of these proteins, without children (dWoc), is essential for viability, associates with chromatin, and prevents telomere fusions [30]–[32]. Interestingly, ZNF198 and ZNF261 are present in transcriptional corepressor complexes that also contain LCH [13], [14], [18], although their functions in transcriptional regulation have not been explored.

Many transcription factors and cofactors are modified by small ubiquitin-like modifier (SUMO). Sumoylation of these factors generally leads to transcriptional repression. For unknown reasons, multiple subunits within a given chromatin-associated complex are often targeted by sumoylation [33], [34]. For example, ZNF198, ZNF262, HDAC1, and LSD1 are known SUMO substrates [33], [35]–[37]. Sumoylation of HDAC1 has been shown to be required for its function. Recent reports have also identified ZNF198 as a non-covalent binding partner for SUMO [38], [39].

In this study, we characterize the function and mechanism of ZNF198-like proteins in regulating the LCH complex. We show that depletion of ZNF198, ZNF261, and ZNF262 by RNA interference (RNAi) in HeLa cells causes derepression of E-cadherin, a known target of LSD1. By contrast, ZNF198-like proteins are not required for the transcriptional repression of several REST-responsive genes that are repressed by LSD1. Consistent with this finding, ZNF198 selectively binds to the LSD1–CoREST–HDAC1 ternary complex and binding of ZNF198 to LCH prevents its interaction with REST. Similar to dWoc, ZNF198 associates with chromatin. Depletion of ZNF198-like proteins weakens the association of LCH with chromatin. Furthermore, sumoylation of HDAC1 decreases its affinity toward CoREST, but enhances its binding to ZNF198. Finally, the tandem repeats of MYM-type zinc fingers of ZNF198 mediate its binding to both LCH and sumoylated HDAC1. Collectively, our results suggest that, unlike the Krüppel-like zinc fingers which bind to DNA, the MYM-type zinc fingers of ZNF198-like proteins mediate multiple protein-protein interactions, maintains the integrity of the LCH complex at non-REST-responsive promoters, and may antagonize SUMO-dependent disassembly of the LCH complex.

## Results

### ZNF198 associates with LSD1, CoREST, and HDACs in human cells

The MYM-type zinc fingers have the CX_2_CX_19–24_[F/Y]CX_3_CX_3_[F/Y] (X is any residue) consensus motif [28]. Five proteins in the human proteome contain tandem repeats of the MYM-type zinc fingers, including ZNF198, ZNF261, ZNF262, ZNF237, and ZNF258 (Figure S1). Some of the MYM zinc fingers in ZNF237 and ZNF258 lack key conserved cysteines (Figure S1), whereas the zinc fingers in ZNF198, ZNF261, and ZNF262 all appear to be intact. ZNF198, ZNF261, and ZNF262 have additional features that differentiate them from ZNF237 and ZNF258. They contain a proline/valine-rich (P/V-rich) domain downstream of the MYM domain. They also contain a domain at their C-terminal region that is predicted by 3D-Jury [40] to have a fold similar to DNA breaking-rejoining enzymes, such as Cre recombinase. The Cre-like domain is also found in several proteins that do not contain MYM zinc fingers (Figure S1).

**Figure 1:**
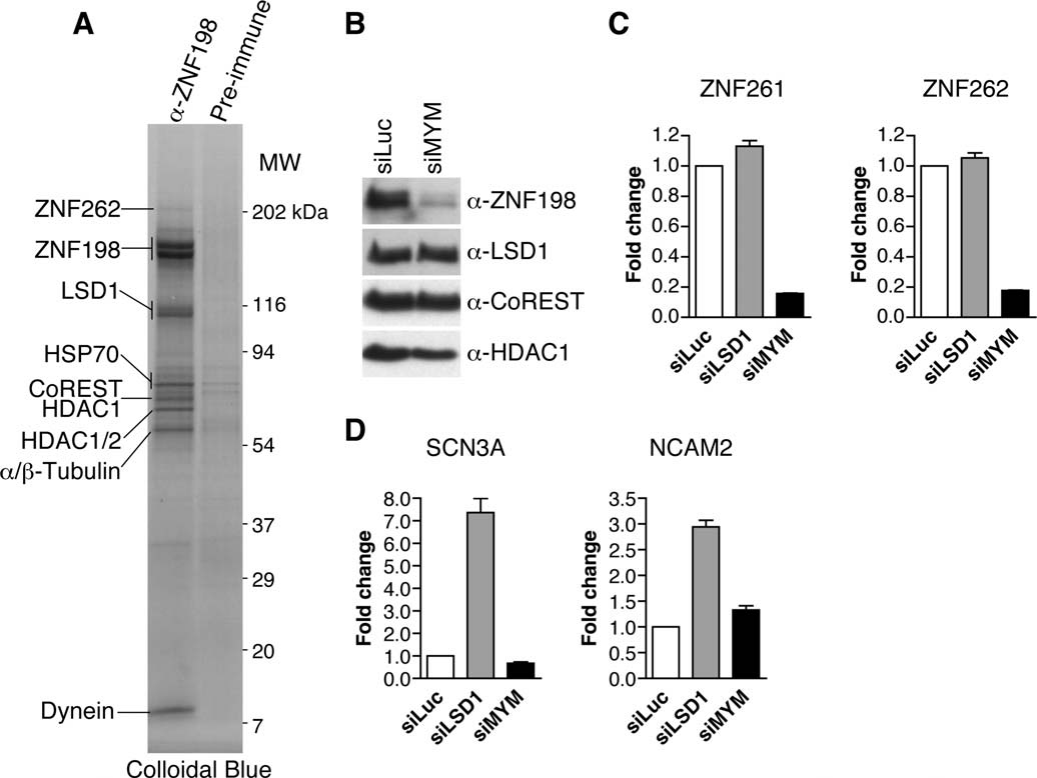
ZNF198-like proteins are not required for the repression of REST-responsive genes. (A) LSD1, CoREST, and HDAC1/2 are major binding partners of ZNF198 in 293 cells. IP of ZNF198 from HEK293 whole cell lysates was separated on SDS-PAGE and stained with Colloidal Blue. Bands were excised and identified by mass spectrometry. (B) HeLa cells were transfected with siRNAs against luciferase (siLuc) or ZNF198, ZNF261, and ZNF262 (siMYM). Cell lysates were blotted with the indicated antibodies. (C & D) U2OS cells were transfected with the indicated siRNAs for three days, followed by quantitative RT-PCR using the indicated primer sets. Cycling-time values were normalized to the housekeeping gene cyclophilin B. Each PCR reaction was performed in triplicate, and error bars indicate the standard deviation of three separate experiments.

ZNF198 and ZNF261 have been shown to be present in several LSD1-containing transcriptional corepressor complexes in sub-stoichiometric amounts [13], [14], [18]. To identify the major ZNF198-interacting proteins in human cells, we immunoprecipitated the endogenous ZNF198 protein from HEK293 and HeLa cells (Figure 1A and data not shown). The ZNF198-binding proteins were detected by Colloidal blue staining followed by mass spectrometry. LSD1, CoREST, and HDAC1/2 were present at near stoichiometric levels. ZNF262 was also present at sub-stoichiometric amounts. Several abundant proteins, including tubulin, Hsp70, and dynein, were also identified in the anti-ZNF198 IP, although they might not be specific ZNF198 interactors. This result indicates that LSD1, CoREST, and HDAC1/2 are major binding proteins of ZNF198 in human cells and confirms earlier findings that have demonstrated the interactions between the LCH complex and proteins containing MYM zinc fingers.

### ZNF198-like proteins are not required for the repression of REST-responsive genes

Through its binding to REST, the LCH complex is recruited to neuron-specific genes and represses their transcription in non-neuronal tissues. We first tested whether ZNF198-like proteins were required for the repression of REST-responsive genes. Because ZNF198, ZNF261, and ZNF262 have all been shown to be associated with LSD1-containing corepressor complexes, we depleted from U2OS and other human cells the three ZNF198-like MYM proteins using RNA intereference (RNAi). As shown in Figure 1B, RNAi against ZNF198, ZNF261, and ZNF262 effectively knocked down the levels of ZNF198 without affecting the levels of LSD1, CoREST, or HDAC1. We did not have antibodies against ZNF261 and ZNF262. However, quantitative RT-PCR analysis (QPCR) confirmed that the siRNAs against these two genes effectively reduced their mRNA levels (Figure 1C). As a comparison, we also depleted LSD1 from human cells using RNAi. Cells transfected with siRNA against luciferase were used as a control. As expected, QPCR analysis revealed that LSD1 RNAi caused an up-regulation of mRNA levels of the known LSD1 target genes, SCN3A and NCAM2 [23] (Figure 1D). By contrast, depletion of ZNF198-like proteins did not significantly alter the mRNA levels of SCN3A and NCAM2 (Figure 1D), suggesting that these proteins were not required for the repression of these putative REST-responsive neuronal genes in non-neuronal tissues.

**Figure 2:**
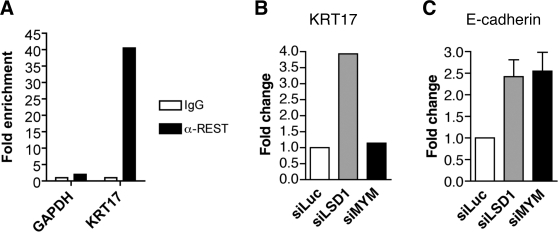
ZNF198-like proteins are required for the repression of E-cadherin. (A) ChIP analysis reveals that REST binds to the promoter of keratin 17 (KRT17). (B) LSD1 is required for the repression of KRT17. U2OS cells were transfected with the indicated siRNAs for three days, followed by quantitative RT-PCR using KRT17 primers. (C) U2OS cells were transfected with the indicated siRNAs for three days, followed by quantitative RT-PCR using the E-cadherin primer set. Cycling-time values were normalized to the housekeeping gene cyclophilin B. Each PCR reaction was performed in triplicate, and error bars indicate the standard deviation of three separate experiments.

To identify additional genes that were repressed by LSD1, we performed microarray analysis of RNA samples from HeLa cells transfected with siRNAs against luciferase or LSD1 (data not shown). Among the genes that were up-regulated by LSD1 RNAi, we confirmed that keratin 17 (KRT17) was a REST-responsive gene, because REST directly bound to the promoter of KRT17 as demonstrated by chromatin immunoprecipitation (ChIP) (Figure 2A). Using QPCR, we confirmed that LSD1 RNAi indeed increased the mRNA levels of KRT17. Depletion of ZNF198-like MYM proteins again had no effect on KRT17 expression (Figure 2B). Therefore, these ZNF198-like proteins do not appear to be required for the repression of REST-responsive genes.

### ZNF198-like proteins are required for the repression of E-cadherin

Corepressor complexes containing LSD1, CoREST, and HDAC1 can be recruited to promoters in REST-independent ways. E-cadherin is a well-characterized gene that is repressed by the CtBP corepressor complex containing LSD1, but E-cadherin is not known to be regulated by REST [16]. We confirmed that E-cadherin was indeed repressed by LSD1 (Figure 2C). RNAi against ZNF198-like MYM proteins also significantly elevated the mRNA level of E-cadherin. Therefore, ZNF198-like proteins are required for the repression of at least some LSD1-repressed genes that are not regulated by REST.

### ZNF198 binds selectively to the LSD1–CoREST–HDAC1 (LCH) ternary complex

**Figure 3:**
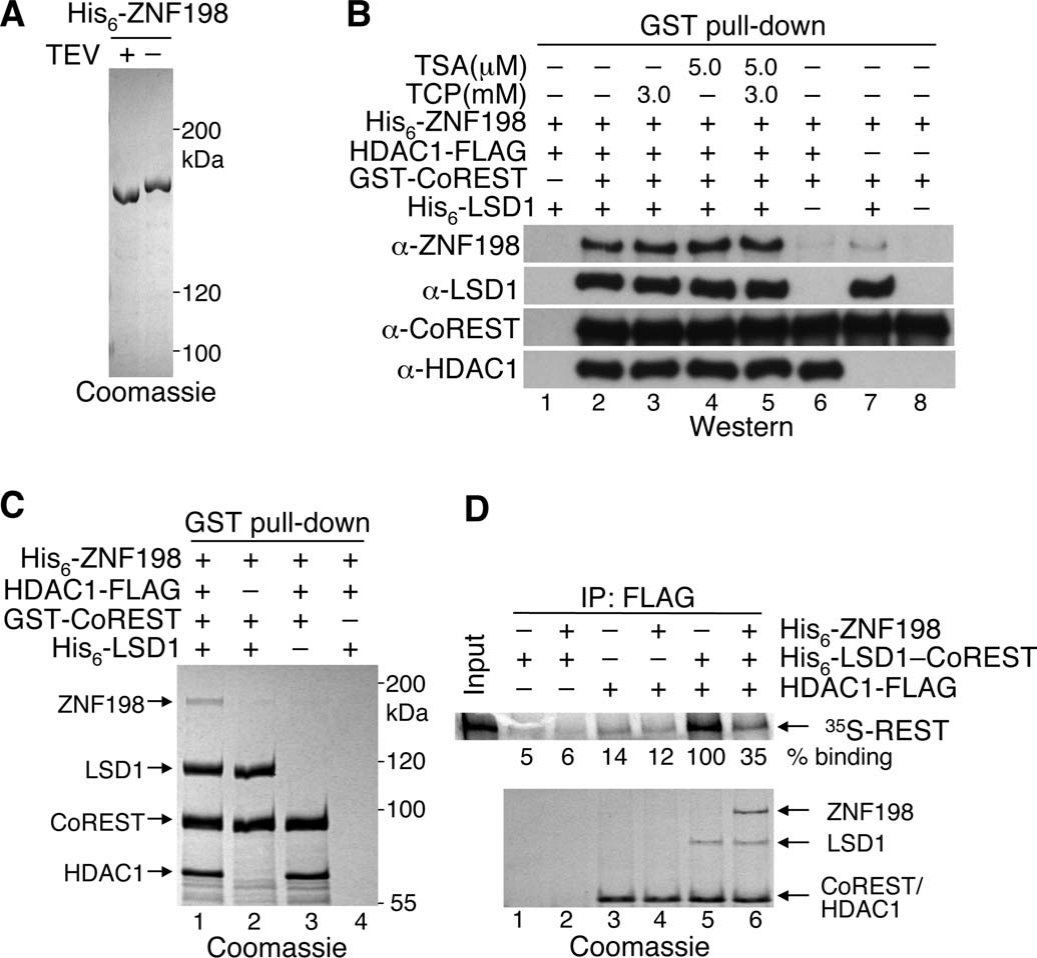
ZNF198 binds preferentially to the intact LSD1–CoREST–HDAC1 (LCH) ternary complex. (A) Recombinant His_6_-ZNF198 purified from Sf9 cells was treated with TEV protease and analyzed by SDS-PAGE followed by Coomassie staining. TEV protease digestion removed the His_6_-tag and caused the protein to migrate faster, thus confirming the identity of the band as His_6_-ZNF198. (B) Recombinant His_6_-ZNF198 (6 µg) was added to glutathione-agarose beads that had been preincubated with 1 µg GST-CoREST, 2 µg HDAC1-FLAG, or 3 µg His-LSD1 as indicated. When indicated, TCP or TSA were present for the entire procedure. After washing, bound proteins were detected by western blotting with the indicated antibodies. (C) GST pull-downs assays were performed as in (B), except that bound proteins were stained with Coomassie blue. The bands belonging to His_6_-ZNF198, His_6_-LSD1, GST-CoREST, and HDAC1-FLAG are labeled. (D) ZNF198 competes with REST for binding to the LCH complex. HDAC1-FLAG (1 µg) and the His_6_-LSD1–His_6_-CoREST binary complex (3 µg) were preincubated with anti-FLAG M2 agarose in the indicated combinations. After washing, ^35^S-REST was added to each binding reaction in the presence or absence of His_6_-ZNF198 (10 µg). Bound REST was detected using a phosphoimager (upper panel). The intensities of REST bands in each reaction were quantified and normalized to lane 5. The values were averages of two experiments. Proteins bound to the anti-FLAG beads were also analyzed by SDS-PAGE and stained with Coomassie blue (lower panel). Note that His_6_-CoREST and HDAC1-FLAG migrate at the same position on the gel.

We next sought to understand the mechanisms by which ZNF198-like MYM-domain proteins regulated the LCH complex. First, we tested whether ZNF198 bound directly to LSD1, CoREST, or HDAC1. To do this, we purified recombinant His_6_-ZNF198, GST-CoREST, His_6_-LSD1, and HDAC1-FLAG (Figure 3A and 3C). Surprisingly, in GST pull-down assays, ZNF198 did not bind efficiently to CoREST alone, LSD1–CoREST, or HDAC1–CoREST (Figure 3B, lanes 6–8 and Figure 3C, lanes 2 and 3). ZNF198, however, bound efficiently to the intact LCH ternary complex (Figure 3B, lane 2; Figure 3C, lane 1). Inhibition of HDAC1 and LSD1 activities using trichostatin A [41] (TSA) and tranylcypromine [42] (TCP), respectively, had no effect on the binding between ZNF198 and LCH (Figure 3B, lanes 3–5). Furthermore, binding of ZNF198 to LCH greatly reduced the binding of REST to LCH (Figure 3D, lanes 5 and 6). This suggests that ZNF198-binding and REST-binding to LCH are mutually exclusive, consistent with our finding that ZNF198 does not appear to be required for the repression of REST-responsive genes.

### ZNF198-like proteins stabilize the LCH complex on chromatin

**Figure 4:**
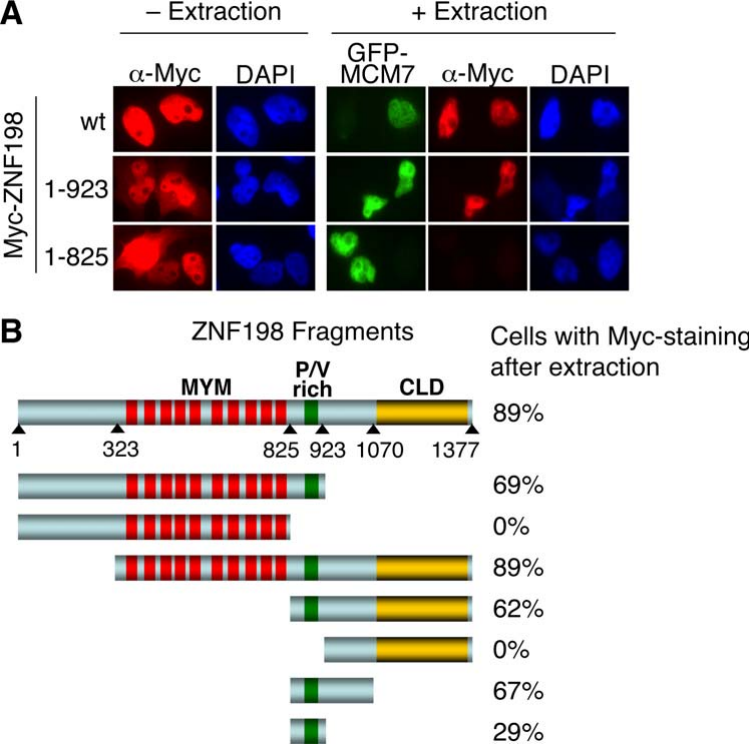
ZNF198 binds to chromatin through its P/V-rich domain. (A) HeLa Tet-on cells were transfected with the indicated Myc-tagged ZNF198 constructs along with GFP-MCM7. Cells were either fixed directly (–Extraction) or extracted prior to fixation (+Extraction) and stained with anti-Myc antibody (red) and DAPI (blue). GFP is shown in green. (B) Summary of the staining data described in (A). The ZNF198 fragments are shown on the left while the percentages of GFP-positive cells that were also Myc-positive after extraction are shown on the right. More than 30 GFP-positive cells from 10 random fields were counted for each fragment. The boundaries of ZNF198 fragments are indicated by triangles.

Binding of ZNF198 to LCH prevents the binding of REST. One possibility is that ZNF198 recruits LCH to specific promoters in a manner similar to REST. However, we were unable to detect high-affinity binding of ZNF198 to DNA *in vitro* (data not shown). Prompted by the finding that the *Drosophila* homolog of ZNF198, Woc, associated with chromatin, we tested whether ZNF198 also bound to chromatin in human cells. We transfected HeLa cells with plasmids encoding Myc-ZNF198 or its fragments. The cells were stained with anti-Myc either with or without extraction before fixation (Figure 4A and 4B). To mark the nuclei of transfected cells after extraction, cells were also co-transfected with a plasmid encoding a known chromatin-bound protein GFP-MCM7. As expected, GFP-MCM7 remained in the nucleus after such extraction, indicating that it was bound to chromatin [43] (Figure 4A). Both the endogenous ZNF198 (data not shown) and the full-length Myc-ZNF198 (Figure 4A) were diffusely nuclear localized and this staining was resistant to extraction, consistent with ZNF198 being bound to chromatin. Analysis of the ZNF198 fragments showed that those fragments lacking the P/V-rich domain were not detected in the nuclei after extraction, even though these nuclei contained GFP-MCM7 and had thus been transfected (Figure 4A and 4B). These data indicate that the P/V-rich domain is required for the association of ZNF198 with chromatin. The P/V-rich region alone, however, bound to chromatin much weaker than the full-length Myc-ZNF198 and its larger fragments (Figure 4B). Therefore, multiple regions in ZNF198 contribute to its association with chromatin.

**Figure 5:**
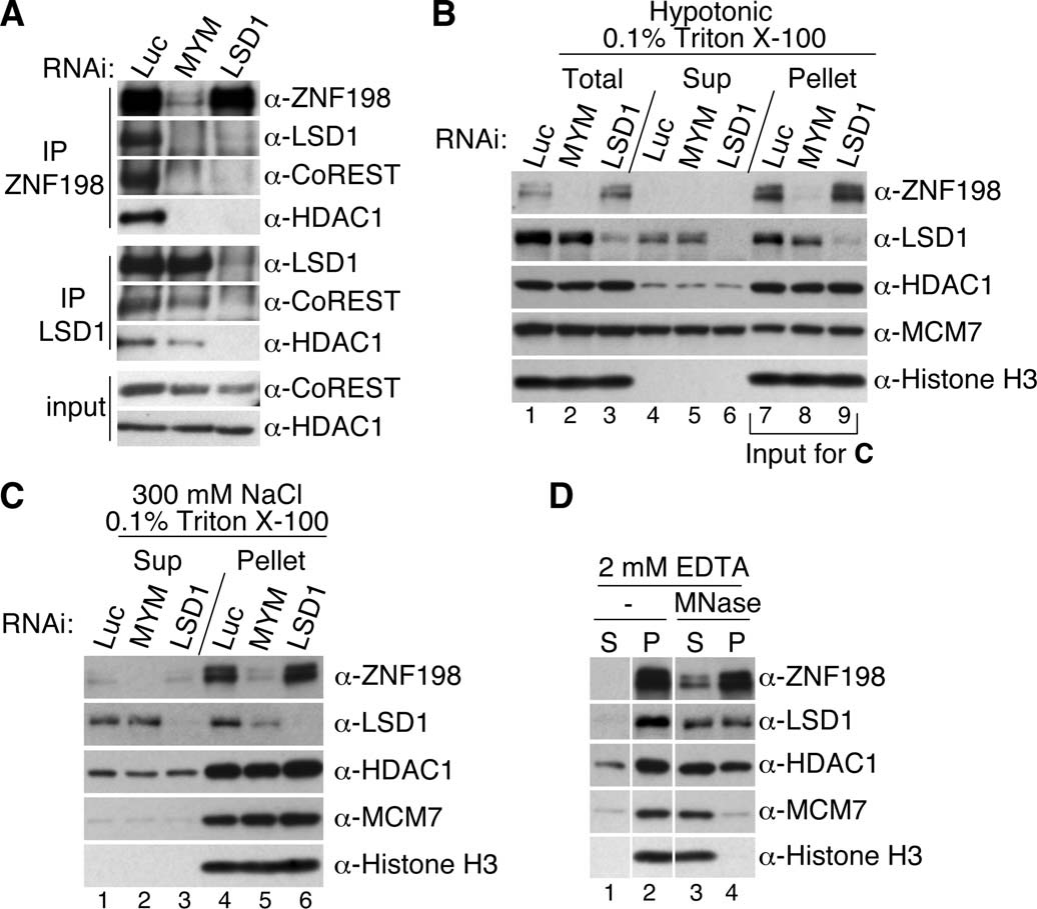
ZNF198-like proteins regulate the chromatin association of LSD1. (A) HeLa Tet-on cells were transfected with the indicated siRNAs: Luc (firefly luciferase), MYM (ZNF198, ZNF261, and ZNF262), or LSD1. Lysates from these RNAi cells were immunoprecipitated with either anti-ZNF198 (top panel) or anti-LSD1 (middle panel). The IPs and the lysates (bottom panel) were blotted with the indicated antibodies. (B) After RNAi of the indicated proteins, nuclear pellets were generated by subjecting HeLa Tet-on cells to hypotonic lysis followed by centrifugation. Normalized samples from each step were subjected to SDS-PAGE and blotted with the indicated antibodies. (C) Nuclear pellets in lanes 7–9 in (B) were subjected to extraction with a high-salt buffer. Normalized samples from each step were blotted with the indicated antibodies. (D) HeLa cells transfected with siLuc were subjected to fractionation as in *B*. The nuclear pellet in lane 7 in (B) was digested with micrococcal nuclease (MNase) followed by extraction with 2 mM EDTA. Supernatants (S) and pellets (P) were blotted with the indicated antibodies.

Formation of an intact LSD1–CoREST–HDAC1/2 complex on chromatin is important for optimal co-repressor activity [19]. Consistent with our *in vitro* finding that ZNF198 interacted specifically with the LCH ternary complex, depletion of LSD1 by RNAi also dramatically reduced the amounts of CoREST and HDAC1 in the α-ZNF198 IPs (Figure 5A, top panel, compare lanes 1 and 3). Through selective binding to the LCH ternary complex, ZNF198 would be expected to stabilize this complex. On the other hand, depletion of ZNF198-like MYM-domain proteins only slightly decreased the association of LSD1 with CoREST and HDAC1 (Figure 5A, middle panel, compare lanes 1 and 2). Thus, ZNF198-like proteins have a role in maintaining the LCH complex, but are not essential for its stability.

We next tested whether depletion of ZNF198-like proteins affected the chromatin binding of LSD1 by subcellular fractionation [44]. The majority of ZNF198 was found in insoluble chromatin fractions (Figure 5B and 5C, compare supernatant and pellets). LSD1 RNAi did not affect chromatin association of ZNF198, suggesting that ZNF198 bound to chromatin independently of LCH (Figure 5B and 5C). By contrast, only a small fraction of LSD1 was associated with chromatin (Figure 5B). RNAi of ZNF198-like proteins reduced the levels of LSD1 in the chromatin fraction by about 2-fold (Figure 5B, compare lanes 7 and 8). This effect was more pronounced after a high-salt extraction of the chromatin fraction (Figure 5C, compare lanes 4 and 5). Importantly, an unrelated chromatin-binding protein MCM7 was unaffected by RNAi against ZNF198-like proteins [45]. Therefore, ZNF198-like proteins are required for efficient chromatin-association of LSD1 and possibly the LCH complex. HDAC1 levels in chromatin fractions, however, were not affected by RNAi against ZNF198-like proteins, presumably because only a fraction of cellular HDAC1 associated with LSD1 and ZNF198 (data not shown).

To further characterize the nature of chromatin association of ZNF198 and LSD1, we digested the chromatin fraction with micrococcal nuclease (MNase) followed by EDTA extraction [44] (Figure 5D). Most of MCM7 and histone H3 were released into the supernatant by MNase digestion (Figure 5D). Although about 50% of HDAC1 and LSD1 and a small fraction of ZNF198 were also released, significant fractions of ZNF198, LSD1, and HDAC1 remained in the pellet. Therefore, at least a portion of the LCH complex associates with nuclease-resistant chromatin materials or nuclear matrix or both.

### Sumoylation of HDAC1 weakens its binding to CoREST but enhances its binding to ZNF198

**Figure 6:**
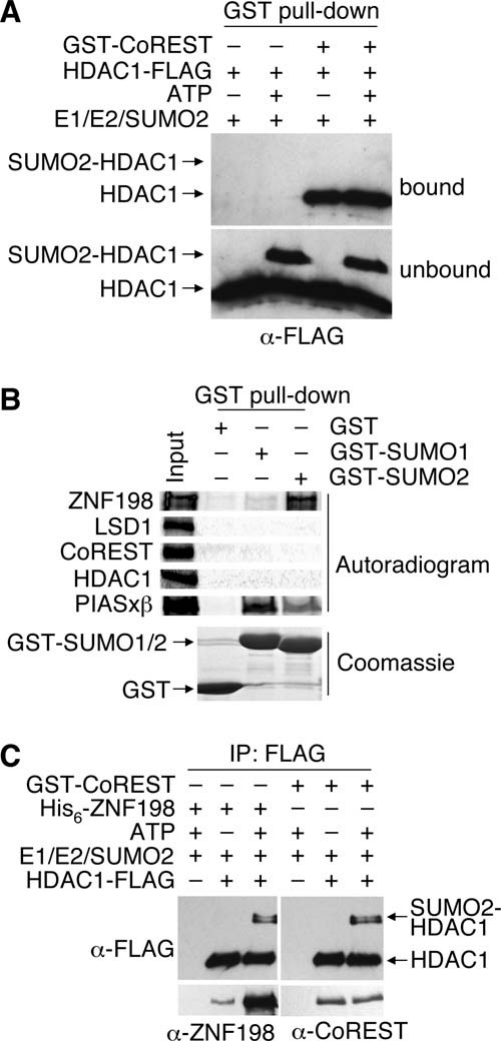
Sumoylation of HDAC1 weakens its interaction with CoREST, but enhances its binding to ZNF198. (A) HDAC1-FLAG (40 ng) was incubated with SUMO2 and sumoylation enzymes with or without ATP. The reaction mixtures were then added to glutathione-agarose beads that had been preincubated with buffer or GST-CoREST (1 µg). After washing, bound (top panel) and unbound (bottom panel) proteins were blotted with anti-FLAG. The sumoylated and un-sumoylated HDAC1 bands are labeled. (B) The indicated ^35^S-labeled *in vitro* translated proteins were incubated with glutathione-agarose beads bound with 10 µg of GST, GST-SUMO1, or GST-SUMO2. The bound proteins were separated by SDS-PAGE, stained with Coomassie blue (bottom panel, a representative image), and analyzed using a phosphoimager (top panel). (C) HDAC1-FLAG (1 µg) bound to anti-FLAG M2 agarose beads was incubated with SUMO2 and sumoylation enzymes with or without ATP. After washing, either His_6_-ZNF198 (6 µg) or GST-CoREST (2 µg) was added to the beads. The bound proteins were blotted with the indicated antibodies.

Both HDAC1 and LSD1 can be sumoylated. Although the function of LSD1 sumoylation has not been established, sumoylation of HDAC1 at its C-terminal region is required for its ability to repress transcription [37]. We were interested in how sumoylation of HDAC1 may affect its interaction with CoREST. Since sumoylation of HDAC1 has been shown to be important for its repressor activity, we hypothesized that SUMO-HDAC1 might show increased affinity for CoREST. We tested this hypothesis by comparing binding of sumoylated and free HDAC1-FLAG to GST-CoREST using GST pull-down assays (Figure 6A). Surprisingly, free HDAC1-FLAG, but not SUMO2-HDAC1-FLAG, bound to GST-CoREST. Thus, sumoylation of HDAC1 inhibits its interaction with CoREST.

Recently, it has been shown that ZNF198 and LSD1 could bind to SUMO non-covalently [38], [39]. To confirm these reports, we tested whether ZNF198, LSD1, CoREST, HDAC1, and PIASxβ bound SUMO non-covalently in GST pull-down assays (Figure 6B). Consistent with previous reports [46], PIASxβ bound to both GST-SUMO1 and GST-SUMO2. By contrast, ZNF198 bound preferentially to GST-SUMO2. We could not detect binding of LSD1, CoREST, or HDAC1 to either GST-SUMO1 or GST-SUMO2. Therefore, our results confirm that ZNF198 binds to SUMO2 non-covalently and suggest that the reported non-covalent binding between LSD1 and SUMO2 in human cell lysates is likely indirect and mediated through ZNF198.

We next tested how sumoylation of HDAC1 might influence its interaction with ZNF198. We performed FLAG IPs with either sumoylated or free HDAC1-FLAG as bait (Figure 6C). ZNF198 exhibited minimal binding to HDAC1 alone. Strikingly, ZNF198 binding to HDAC1 was greatly enhanced by HDAC1 sumoylation, even though a minor fraction of HDAC1 was sumoylated in this particular assay. As a control, this minimal sumoylation of HDAC1 did not perturb its binding to CoREST, because the majority of HDAC1 remained unsumoylated and retained the ability to bind to CoREST (Figure 6C). Thus, sumoylation of HDAC1 weakens its binding to CoREST, but enhances its binding to ZNF198 *in vitro*.

Many non-covalent binding partners of SUMO are efficiently sumoylated *in vitro* [46], [47]. Consistent with a previous report [35], ZNF198 was efficiently sumoylated *in vitro* (Figure S2A). Many efficient SUMO substrates with SUMO binding capacity, such as RanBP2 and PIASxβ, are also SUMO ligases [46], [47]. ZNF198, however, failed to stimulate the sumoylation of either LSD1 or HDAC1 *in vitro* (Figure S2B). Therefore, we do not have evidence to suggest that ZNF198 functions as a SUMO ligase.

### MYM-type zinc fingers of ZNF198 mediate its interactions with the LCH complex and sumoylated HDAC1

**Figure 7:**
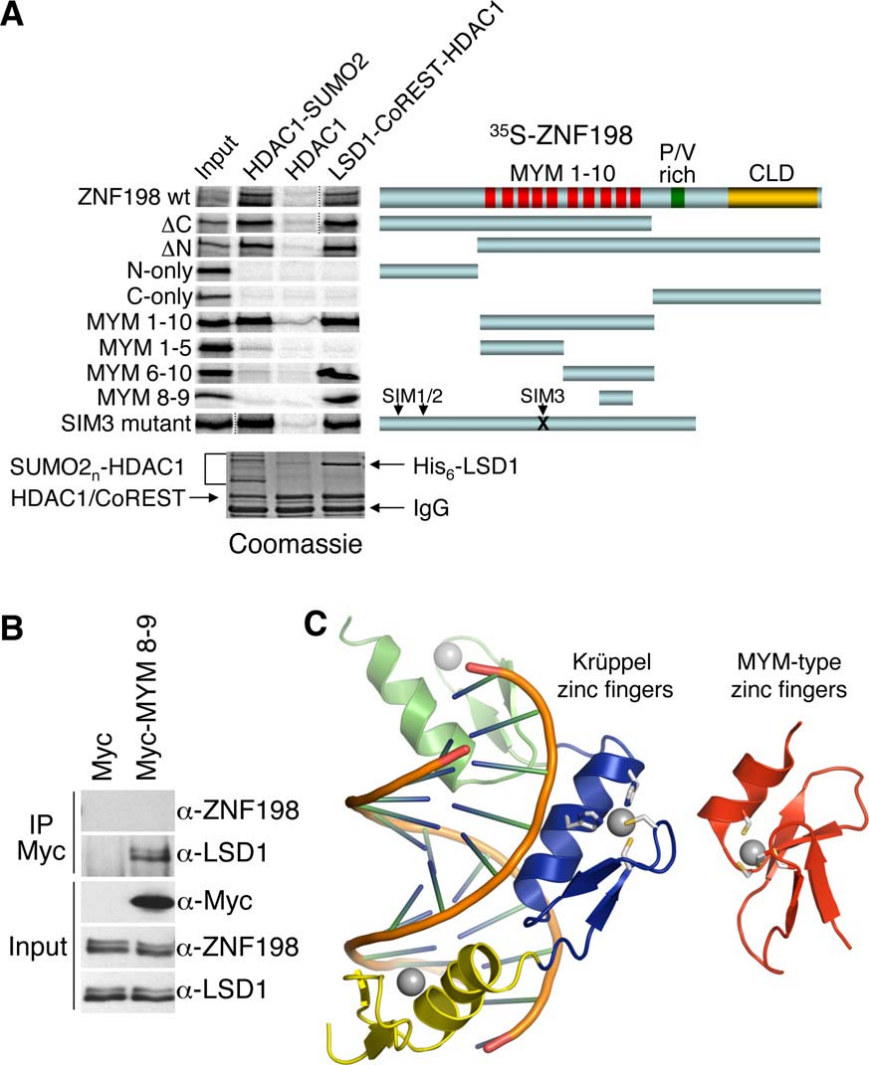
MYM-type zinc fingers of ZNF198 mediate its binding to the LCH complex and HDAC1-SUMO2. (A) ^35^S-labeled ZNF198 fragments and mutants were incubated with anti-FLAG beads that had been preincubated with HDAC1-SUMO2, HDAC1, or the LSD1–CoREST–HDAC1 complex. After washing, the bound proteins were visualized by autoradiography and Coomassie blue staining (bottom panel). Three putative SUMO-interacting motifs (SIMs) are indicated. The SIM3 mutant contains V483A, L484A, and V485A mutations. (B) HeLa Tet-on cells were transfected with the indicated constructs for 24 hours. Lysates and the Myc IPs of the transfected cells were blotted with the indicated antibodies. (C) Ribbon drawings of three Krüppel-like zinc fingers bound to DNA (PDB ID: 1ZAA; left) and a MYM-type zinc finger from ZNF237 (PDB ID: 2DAS; right). Zinc ions are shown as spheres while zinc-binding ligands are shown in sticks.

We next mapped the regions of ZNF198 required for binding to the ternary LCH complex or HDAC1-SUMO2. We used recombinant FLAG-tagged HDAC1-SUMO2, HDAC1, or LSD1–CoREST–HDAC1 as baits. As expected, full-length ZNF198 bound to both SUMO2-HDAC1 and LSD1–CoREST–HDAC1, but not to HDAC1 alone (Figure 7A). Analysis of a series of truncation mutants of ZNF198 revealed that the central region of ZNF198 containing the 10 tandem MYM-type zinc fingers was necessary and sufficient for binding to both SUMO2-HDAC1 and LSD1–CoREST–HDAC1 (Figure 7A). Further mapping revealed that a small ZNF198 fragment containing two zinc fingers, MYM8-9, was sufficient for binding to the LCH complex *in vitro* (Figure 7A). MYM8-9 could also be co-immunoprecipitated with LSD1 from HeLa cell lysates (Figure 7B), although it was unclear whether it bound LSD1 as efficiently as the wild-type ZNF198. By contrast, efficient binding of ZNF198 to SUMO2-HDAC1 required larger fragments of ZNF198 containing most of its MYM-type zinc fingers (Figure 7A). Notably, ZNF198 has three putative SUMO interaction motifs (SIMs) [38]. Deletion of the N-terminal region containing SIM1 and SIM2 or mutation of SIM3 did not affect the binding of ZNF198 to HDAC1-SUMO2 (Figure 7A), indicating that none of these putative SIM motifs of ZNF198 are important for HDAC1-SUMO2 binding. Thus, zinc fingers 8 and 9 of ZNF198 mediate its binding to LCH, whereas additional zinc fingers are required for the binding of ZNF198 to HDAC1-SUMO2.

## Discussion

### Functions of ZNF198-like proteins in transcriptional repression

Consistent with a role of ZNF198 in transcriptional regulation, several well established transcriptional repressors, including LSD1, CoREST and HDAC1/2, are major binding proteins of ZNF198 *in vivo*. Interestingly, ZNF198 only interacts with the intact LSD1–CoREST–HDAC1 (LCH) ternary complex. Moreover, ZNF198- and REST-binding to LCH are mutually exclusive. Expectedly, ZNF198-like proteins are dispensable for the LSD1-mediated repression of REST-responsive neuron-specific genes in non-neuronal cell lines, such as HeLa and U2OS. This created a serious challenge for our studies on ZNF198, because most known LSD1-repressed genes are REST-responsive. Despite this difficulty, we managed to show that ZNF198-like proteins are required for the transcriptional repression of E-cadherin, a well-established LSD1-repressed gene that is not REST-responsive. Identification of additional genes whose repression requires ZNF198-like proteins is needed to fully understand their biological functions.

We have further shown that ZNF198 binds directly to chromatin in part through its proline/valine-rich domain. Depletion of ZNF198 reduces the amount of chromatin-bound LSD1 as revealed by subcellular fractionation experiments. Thus, one possible mechanism by which ZNF198-like proteins facilitate the functions of the LSD1–CoREST–HDAC1-containing corepressor complexes is to recruit or stabilize the LSD1–CoREST–HDAC1 ternary complex on chromatin. Because we have so far failed to detect ZNF198 at specific promoters using ChIP, it remains to be determined whether ZNF198-like proteins are required for the stable association of the LCH complex at specific promoters. It will also be interesting to test whether, in addition to tethering the LCH complex to chromatin, ZNF198 directly facilitates the demethylation or deacetylation or both of nucleosomes by the LCH complex.

### ZNF198 and sumoylation of HDAC1

Sumoylation of HDAC1 is required for its function in transcriptional repression [37], [48]. For example, cells stably expressing wild-type HDAC1, but not its sumoylation-deficient mutant, show cell cycle defects [37]. The sumoylation-deficient mutant of HDAC1 also shows lower deacetylase activity [37], [48], suggesting that sumoylation is required for the full activation of the catalytic activity of HDAC1. We show that HDAC1 sumoylation inhibits its binding to CoREST. Our finding is consistent with previous findings that the C-terminus of HDAC1 mediates its interactions with cofactors [49] and mutation of the HDAC1 sumoylation sites does not disrupt cofactor binding [37].

HDAC1 and LSD1 exhibit positive cooperativity in deacetylating and demethylating nucleosomes. CoREST bridges the interaction between HDAC1 and LSD1. Disruption of the HDAC1–CoREST interaction by HDAC1 sumoylation is thus expected to abolish the positive cooperativity between HDAC1 and LSD1. Paradoxically, sumoylation of HDAC1 is required for its function and possibly activity. Our finding that sumoylation of HDAC1 enhances its binding toward ZNF198 provides one possible way to resolve the paradox of HDAC1 sumoylation. Through its abilities to bind multiple components of the LCH complex and to sumoylated HDAC1, ZNF198 may antagonize the disruption of the HDAC1–CoREST interaction by HDAC1 sumoylation and preserve the integrity of the LCH complex on chromatin. The physiological significance of these *in vitro* findings remains to be established.

### MYM-type zinc fingers as protein-protein interaction modules

ZNF198 has been proposed to recruit transcriptional corepressors to specific promoters through sequence-specific DNA-binding, a function that is analogous to REST. This hypothesis largely stems from the fact that both ZNF198 and REST contain tandem zinc fingers and bind to the LCH complex [18], [20], [21]. Furthermore, ZNF198 competes with REST for CoREST-binding. However, the tandem zinc fingers of REST are Krüppel-like zinc fingers that mediate sequence-specific DNA-binding to RE1-elements in promoters [50], [51]. Structure comparison reveals that the MYM-type zinc fingers in ZNF198 have a fold that is distinct from that of the Krüppel-like zinc fingers (Figure 7*C*). In particular, the long α-helix that mediates DNA binding in Krüppel-like zinc fingers [52] is much shorter in the MYM-type zinc fingers. Therefore, the MYM-type zinc fingers are unlikely to bind to DNA in a manner similar to the Krüppel-like zinc fingers. In fact, a search using the Dali server (http://www.ebi.ac.uk/dali) revealed that the fold of MYM-type zinc fingers is most related to that of the LIM (Lin11, Isl-1 & Mec-3) domain. LIM domains are also often found in tandem repeats and mediate protein-protein interactions [53]. The structural similarity between the MYM and LIM domains further supports a role for MYM-type zinc fingers in mediating protein-protein interactions.

**Figure 8:**
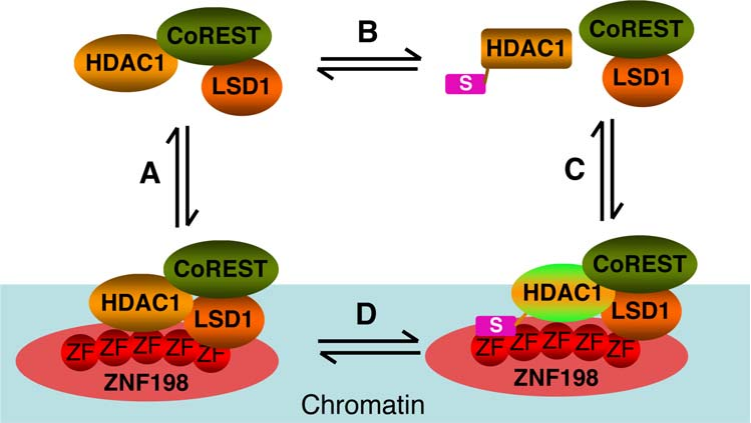
Proposed mechanisms by which ZNF198 regulates the LSD1–CoREST–HDAC1 complex. See DISCUSSION for details.

In conclusion, we have revealed a functional requirement of ZNF198-like proteins in transcriptional repression of LSD1-repressed genes that are not REST-responsive. Our results further suggest the following model to explain the mechanism by which ZNF198 promotes the functions of LSD1 (Figure 8). In this model, ZNF198 binds to chromatin and recruits the LSD1–CoREST–HDAC1 ternary complex to chromatin. The MYM-type zinc fingers of ZNF198 mediate its interactions with the LCH complex and with sumoylated HDAC1. Through its ability to engage in multiple protein-protein interactions, ZNF198 stabilizes the LCH complex on chromatin and possibly prevents the dissociation of HDAC1 from the complex that is triggered by HDAC1 sumoylation.

## Materials and Methods

### Protein expression and purification

SUMO-related constructs and protein purification were described previously [33]. The coding regions of CoREST and LSD1 were amplified from human fetal thymus cDNA library (BD Biosciences) and ZNF198 was amplified from a purchased cDNA plasmid (Open Biosystems) by PCR. The PCR products were digested and ligated into appropriate expression vectors. Similar methods were used to construct plasmids encoding various ZNF198 fragments. The pSC-β-REST construct was a gift from Jenny Hsieh.

The full-length His_6_-LSD1, His_6_-LSD1–His_6_-CoREST, or His_6_-ZNF198 were expressed in Sf9 insect cells and purified using a combination of Ni^2+^-Sepharose (Amersham) affinity chromatography and ion exchange chromatography (Resource Q, Amersham). HDAC1-FLAG was purified with M2 agarose beads (Sigma) from Sf9 cell lysates and eluted with the FLAG peptide. GST-CoREST, GST-SUMO1, and GST-SUMO2 were purified from bacterial lysates using glutathione-agarose resin (Amersham). Proteins were stored in buffers containing 50 mM Tris-HCl, pH 8.1, 50–200 mM KCl, 10% glycerol, and 1 mM DTT.

### Cell culture and transfections

HeLa Tet-on (BD Biosciences) and U2OS cells were grown in DMEM (Invitrogen) supplemented with 10% fetal bovine serum, 2 mM L-glutamine, and 100 µg/ml penicillin and streptomycin at 37°C and 5% CO_2_. DNA and siRNA transfections were performed with the effectene reagent (Qiagen) and Lipofectamine RNAiMax reagent (Invitrogen), respectively, according to manufacturer's protocols. The siRNA sequences are: 5′- GGCCUAGACAUUAAACUGA-3′ (LSD1), 5′-GGGCCAGACAGCUUAUCAA-3′ (ZNF198), 5′-GACCCUGUGUAAGAACUUU-3′ (ZNF261), 5′-CACCACCACUAGUAAAGAU-3′ (ZNF262).

### Cell fractionation

Lysates of HeLa Tet-on cells (2×10-cm plates) transfected with siRNAs for 48 hrs were fractionated as described [44] with one significant modification. We included an additional high-salt extraction step using buffer C (10 mM HEPES, pH 7.9, 10 mM KCl, 300 mM NaCl, 1.5 mM MgCl_2_, 25% glycerol, 0.1% Triton X-100, 1 mM DTT, 10 µg/ml protease inhibitor cocktail, and 0.4 mM PMSF).

### Immunofluorescence

HeLa Tet-on cells transfected with various plasmids were either extracted as previously described [43] and then fixed with 4% paraformaldehyde or directly fixed. All samples were then permeabilized with 0.1% Triton-X100 in PBS, and incubated with 1 µg/ml of anti-Myc (9E10, Roche). After washing, fluorescent secondary antibodies (Molecular Probes) were added at 1∶500 dilutions. The cells were again washed three times with PBS, counter-stained with DAPI, and viewed using a 63× objective on a Zeiss Axiovert 200 M microscope. Images were acquired using the Intelligent Imaging software, and pseudo-colored in Adobe Photoshop.

### Antibodies, immunoprecipitation, and immunoblotting

Rabbit polyclonal antibodies against ZNF198 and LSD1 were generated using a ZNF198 fragment (residues 923–1377) and an LSD1 fragment (residues 171–852) as the antigens at Zymed and Yenzym, respectively. The following antibodies were purchased from Upstate: α-HDAC1 (05-614), α-CoREST (07-455). Large-scale immuno-purification of ZNF198-containing protein complexes was performed as described [54]. For IP and western experiments, HeLa Tet-on cells from a 10-cm dish were washed and harvested in cold PBS 2 days after transfection and lysed in 1 ml of buffer C supplemented with 0.5 µM okadaic acid. Antibodies immobilized on Affi-prep protein A beads were incubated with the lysates for 2 hrs at 4°C. After washing, the beads were dissolved in SDS sample buffer and analyzed by SDS-PAGE following by immunoblotting. For immunoblotting, crude sera were used at 1∶1000 dilution while purified antibodies were used at a final concentration of 1 µg/ml.

### *In vitro* binding and sumoylation assays

*In vitro* transcription and translation and *in vitro* sumoylation assays were performed as previously described [33]. For binding assays, HDAC1-FLAG, GST-CoREST, or GST-SUMO1/2 proteins together with other proteins were incubated with 5–10 µl M2 agarose (Sigma) or glutathione-sepharose 4B (Amersham) beads in 50 µl binding solution (TBS supplemented with 0.05% Tween-20 and 1 mM DTT) for 1 hr. After washing, the beads were then incubated in 50 µl blocking solution (TBS supplemented with 0.05% Tween-20, 5% dry milk, 1 mM DTT) for 1 hr at room temperature. The appropriate recombinant proteins or 5 µl ^35^S-labeled *in vitro* translated proteins were incubated with the beads for 1 hr at room temperature. Beads were then washed four times with the binding solution, boiled in SDS sample buffer, and subjected to SDS-PAGE followed by Coomassie Blue staining and autoradiography. For binding reactions containing ZNF198, 100 µM ZnCl_2_ was included in all buffers.

### Reverse transcription and quantitative PCR

RNA from U2OS cells grown on 6-well plates and transfected with siRNAs was extracted using TriZOL reagent (Invitrogen) followed by RNAeasy RNA purification kit (Qiagen). RNA was then subjected to DNase digestion and inactivation followed by reverse transcription using random hexamers as primers. 2.5 µl of this cDNA was then used for quantitative PCR in 20 µl reactions using a 2× SYBR Green mix (Bio-Rad). The primers used were: SCN3A-Fwd (5′-ATGCTGGGCTTTGTTATGCT-3′), SCN3A-Rev (5′-TGGCTTGGCTTCAGTTTTCT-3); Cyclophilin B-Fwd: (5′-GGAGATGGCACAGGAGGAA-3′), Cyclophilin B-Rev (5′-GCCCGTAGTGCTTCAGTTT-3′); E-cadherin-Fwd (5′-GGATGACACAGCGTGAGAGA-3′), E-cadherin-Rev (5′-ACAGGATGGCTGAAGGTGAC-3′), NCAM2-Fwd (5′-CACGTTCACTGAAGGCGATA-3′), NCAM2-Rev (5′-GCTGCCCTTTGACTTCGATA-3′). KRT17-Fwd (5′-ATGCAGGCCTTGGAGATAGA-3′), KRT17-Rev (5′-AGGGATGCTTTCATGCTGAG-3′). All primers were validated as described [55].

### Chromatin immunoprecipitation (ChIP)

ChIP experiments were performed as described [56]. About 1×10^7^ HeLa Tet-on cells was used for each IP. Quantitative PCR was performed with 2.5 µl of eluted DNA, using the following primers: KRT17 ChIP-Fwd (5′-GGATAGGCTCTCGGTCTCCT-3′), KRT17 ChIP-Rev (5′-GTCTTTCACCCCACACTGCT-3′), GAPDH ChIP-Fwd (5′-TGTGCCCAAGACCTCTTTTC-3′), GAPDH ChIP-Rev (5′-TATTGAGGGCAGGGTGAGTC-3′).

## Supporting Information

Figure S1 Domain architecture of MYM domain-containing proteins. The following color schemes from this illustration are used throughout the manuscript: MYM-type zinc fingers (MYM, red); proline/valine-rich domain (P/V-rich, green); Cre-like domain (CLD, gold); glutamine-rich domain (Q-rich, gray); potassium-tetramerization domain (K-tetra, black); and transposase-like domain (teal). Non-cysteine residues at zinc-coordinating positions in certain MYM domains are indicated by asterisks. Scale bar indicates 100 amino acids. The Cre-like domain of KCTD1 was used for 3D-Jury analysis (http://Bioinfo.Pl/Meta).
(0.50 MB TIF)

Figure S2 ZNF198 does not stimulate the sumoylation of HDAC1 or LSD1. (A) 35S-labeled in vitro translated ZNF198 was incubated with sumoylation enzymes (E1 and E2) and ATP in the presence or absence of SUMO2. The reaction mixtures were separated by SDS-PAGE and analyzed using a phosphoimager. The bands of unmodified and sumoylated ZNF198 are labeled. (B) Mixtures of His-LSD1 (300 ng), HDAC1-FLAG (300 ng), and GST-CoREST (100 ng) were subjected to in vitro sumoylation reactions in the presence or absence of His-ZNF198 (1–2 µg). The reaction mixtures were blotted with anti-FLAG (top panel) or anti-LSD1 (bottom panel).
(0.33 MB TIF)
