# Supplementary figures and images for: ZNF198 Stabilizes the LSD1–CoREST–HDAC1 Complex on Chromatin through Its MYM-Type Zinc Fingers
Source: PLoS One. 2008 Sep 22;3(9):e3255. doi: 10.1371/journal.pone.0003255 (PMC2532748; doi:10.1371/journal.pone.0003255)

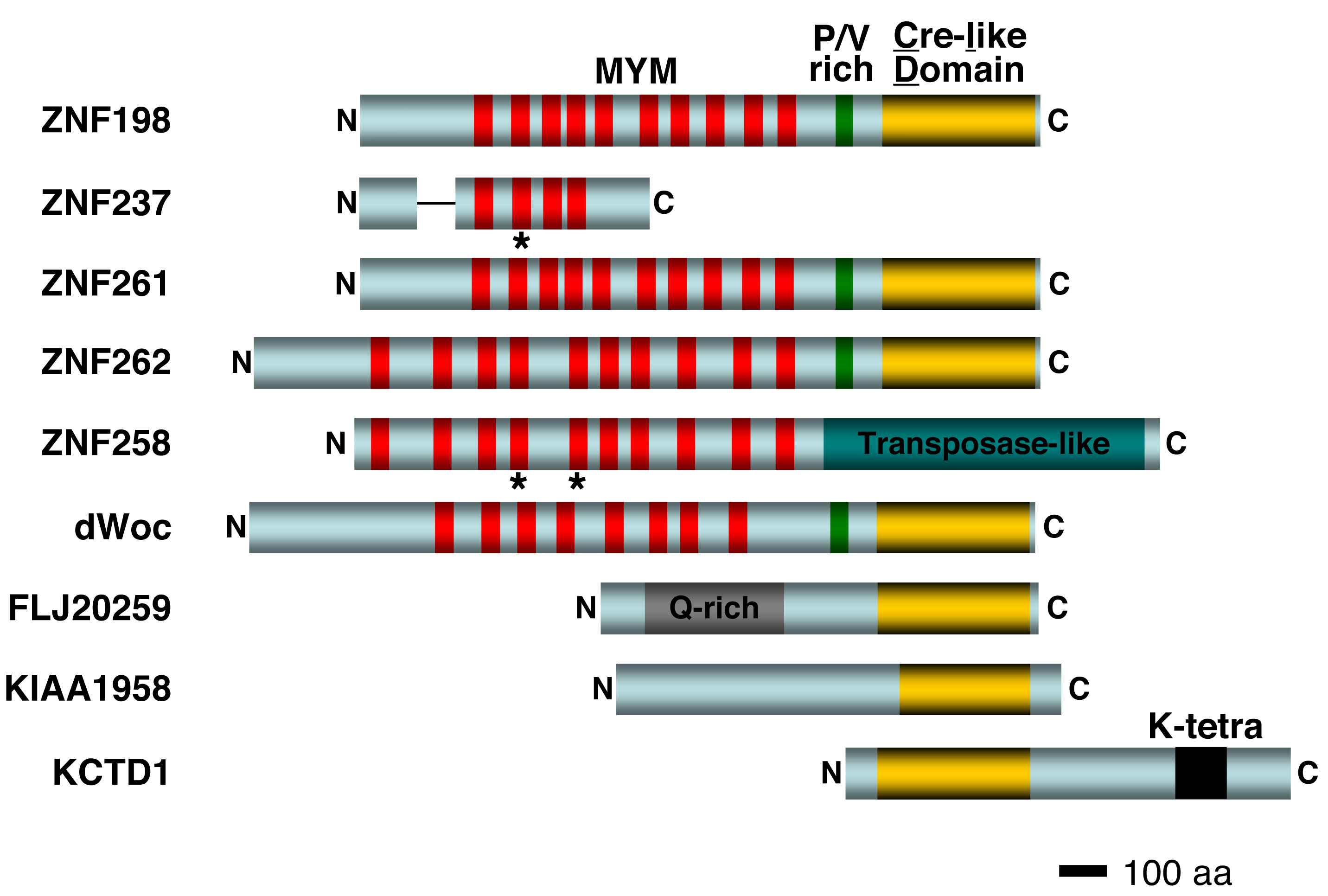

Supplement: Figure S1 — Domain architecture of MYM domain-containing proteins. The following color schemes from this illustration are used throughout the manuscript: MYM-type zinc fingers (MYM, red); proline/valine-rich domain (P/V-rich, green); Cre-like domain (CLD, gold); glutamine-rich domain (Q-rich, gray); potassium-tetramerization domain (K-tetra, black); and transposase-like domain (teal). Non-cysteine residues at zinc-coordinating positions in certain MYM domains are indicated by asterisks. Scale bar indicates 100 amino acids. The Cre-like domain of KCTD1 was used for 3D-Jury analysis (http://Bioinfo.Pl/Meta). (0.50 MB TIF) [file pone.0003255.s001.tif]

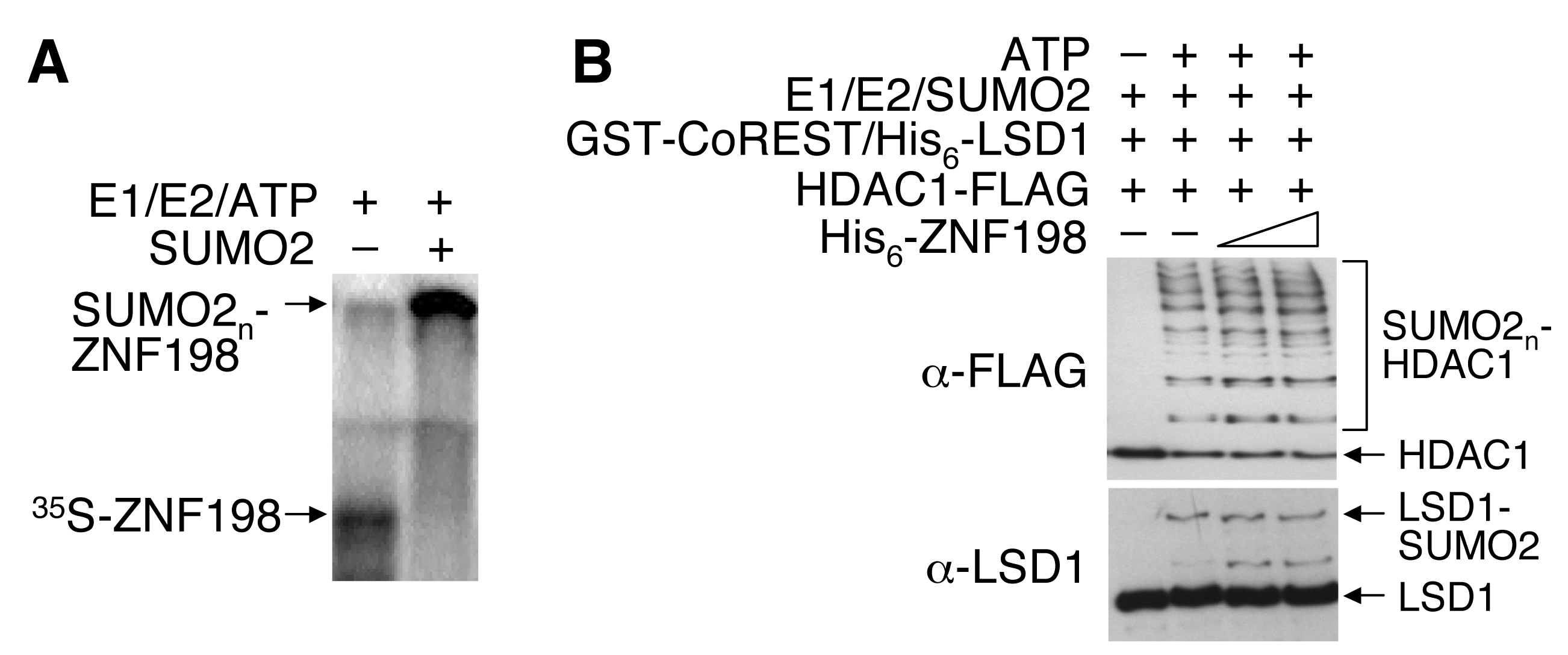

Supplement: Figure S2 — ZNF198 does not stimulate the sumoylation of HDAC1 or LSD1. (A) 35S-labeled in vitro translated ZNF198 was incubated with sumoylation enzymes (E1 and E2) and ATP in the presence or absence of SUMO2. The reaction mixtures were separated by SDS-PAGE and analyzed using a phosphoimager. The bands of unmodified and sumoylated ZNF198 are labeled. (B) Mixtures of His-LSD1 (300 ng), HDAC1-FLAG (300 ng), and GST-CoREST (100 ng) were subjected to in vitro sumoylation reactions in the presence or absence of His-ZNF198 (1–2 µg). The reaction mixtures were blotted with anti-FLAG (top panel) or anti-LSD1 (bottom panel). (0.33 MB TIF) [file pone.0003255.s002.tif]
